# Point density exclusion mapping—A useful tool for mapping arrhythmias arising from the endocavitary structures

**DOI:** 10.1002/joa3.12606

**Published:** 2021-07-23

**Authors:** Mukund A. Prabhu, Sabari Saravanan, Ajit kumar Valaparambil, Narayanan Namboodiri

**Affiliations:** ^1^ Kasturba Medical College Manipal Academy of Higher Education Manipal India; ^2^ Abbott EPHF Chennai India; ^3^ Sree Chitra Tirunal Institute for Medical Sciences and Technology Trivandrum India

**Keywords:** endocavitary structures, papillary muscles, point density exclusion mapping, ventricular tachycardia

## Abstract

Ventricular tachycardia arising from the papillary muscles and other endocavitary structures are preferably ablated under intracardiac echocardiographic (ICE) guidance whenever feasible. However, the availability, need of trained operators, and the expenses involved restrict the routine use of ICE in many cath labs. Point density exclusion (PDX) mapping is a simple technique that doesn't demand any additional expense or tool apart from the routine electroanatomical mapping and thus can be widely applied in mapping of arrhythmias arising from endocavitary structures. The following report describes such a case and explains the method of performing PDX mapping

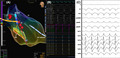

## 1 INTRODUCTION

Intracardiac echocardiography (ICE) is a useful modality for imaging endocavitary structures of the ventricles and has become the standard accessory tool while ablating arrhythmias arising from these structures. However, this is not freely available in most of the electrophysiological (EP) labs globally and significantly adds to the procedural cost thus hampering its utility. Point density exclusion (PDX) mapping, a recently described simple technique, helps to visualize the endocavitary structures like the papillary muscle (PM), needs no additional tools apart from high density geometry and activation mapping, and can be done using any electroanatomical mapping (EAM) system.^1^ The following report illustrates the use of PDX mapping in precise mapping and successful ablation of a ventricular tachycardia (VT) arising from the PM (PM‐VT).

## 2 CASE DETAILS

**FIGURE 1:**
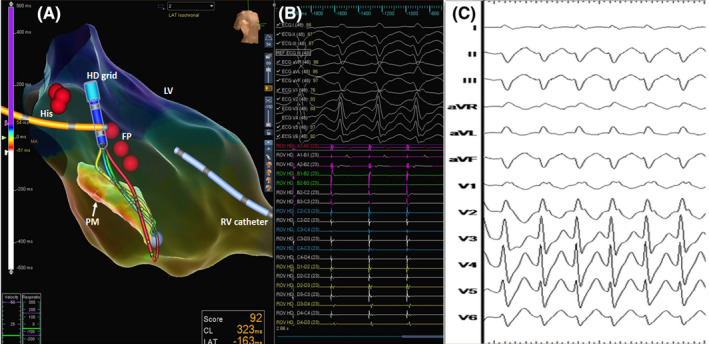
Panel A is the color‐coded activation map showing the earliest activation and a focal spread from the posteromedial papillary muscle. The Advisor HD grid catheter is in good contact with the muscle as deduced form the angle between the catheter shaft and the grid. The image shows the posteromedial PM and is derived from the usual LV map by applying PDX mapping. The red tags represent the His and the posterior fascicular potentials (FP). The score shown in the inset below represents that of the match during pace mapping (shown in Panel B). The ECG during pace map that shows 92% match with the clinical VT and the site of pacing is marked by the violet dot (A1B1 bipole of the HD grid catheter). The pacing site was close to but not exactly at the site of earliest activation. Panel C shows the 12‐lead ECG of the tachycardia for comparison. Paper speed in both the panels B & C is 50 mm/s

A 48‐year‐old gentleman with mitral valve prolapse and moderate mitral regurgitation had recurrent episodes of VT with one of the episodes needing electrical cardioversion. His cardiac magnetic resonance imaging showed no myocardial enhancement and normal biventricular function. He was planned for electrophysiological study and ablation for the VT. The electrocardiogram suggested a VT arising from the posteromedial PM and the procedure was done using the Ensite Precision system (Abbott, Abbott Park,55 Illinois) for EAM. The basal intracardiac intervals were normal, and the tachycardia was consistently inducible by burst pacing from the right ventricular apex but not with the extra‐stimulus protocol. The left ventricular (LV) geometry was created using the Advisor HD grid mapping catheter (Abbott, Abbott Park,55 Illinois) and the Ensite Precision system, using the retroaortic approach. The activation map revealed a focal mechanism with a centrifugal spread of activation from the site of the earliest activation in the mid‐level of the posteroseptal LV aspect. The local electrogram here had high frequency pre‐potentials that preceded the surface QRS by 30 ms, and there was an excellent (92%) match during pace‐mapping from this region (Figure 1). The Advisor HD grid was exchanged for a 4 mm irrigation‐tipped Flexibility catheter (Abbott, Abbott Park,55 Illinois), and activation and pace mapping were done to reconfirm the site of origin. Subsequently, point density exclusion (PDX) technique was applied to display the geometry of the PM and the earliest activation was observed to be arising from this structure. Radiofrequency ablation was performed at this site and after a few runs of automaticity, the VT became non‐inducible. A transthoracic echocardiogram performed with the ablation catheter in situ confirmed its location on the posteromedial PM. The procedure time was 127 min, and the fluoroscopic time was 6 minutesand 27 seconds.

## 3 PDX MAPPING

The PDX mapping has several advantages over ICE‐guided imaging in EP procedures. Firstly, the acquisition of the geometrical points and activation mapping from the endocavitary structure is simultaneous and from the same catheter unlike in ICE. Secondly, there is no need of any additional hardware/software for employing this technique apart from the conventional EAM, thus improving the feasibility and reducing the procedural cost so that it can potentially be applied in any EP lab with EAM facility. Thirdly, the procedural time is less than when using ICE for imaging/image integration. Fourthly, the technique is platform neutral and can be used with any of the EAM systems.

The use of a contact force sensing catheter for ensuring a good contact with the endocavitary structure, though preferable, is not mandatory as illustrated in the current case. The near‐perpendicular bending of the electrode grid and the shaft of the Advisor HD grid catheter indicates good tissue contact, and only such sites were used to derive the PDX mapping in our case.

However, there are also a few potential concerns while using PDX mapping as a replacement for ICE guided ablation. The catheter stability needs to be ensured as the PDX mapping will not be able to detect catheter movements and the resultant inaccuracy. The utility of ICE for visualizing lesion formation, and as a guide for visualizing structures like the interatrial septum, pulmonary veins, and detecting pericardial effusion obviously cannot be replaced by PDX mapping. Also, PDX mapping is a post‐acquisition processing technique that is applied after obtaining the entire geometrical and the activation points and is heavily dependent on a skillful technical assistant.

## 4 THE TECHNIQUE FOR PDX MAPPING

In the standard EAM, the inner geometrical points are automatically excluded as more outer points are added as the mapping catheter roves. Thus, endocavitary structures would be excluded from the final representation of the geometry. In PDX mapping, these internal points and the corresponding activation timing are manually included after the acquisition of a high‐density geometry and thus the visualization of the endocavitary structures is made possible. For ensuring accuracy, including only the inner points with a contact force of at least 8 gms is recommended though other surrogates may be used as in the current case.

**FIGURE 2:**
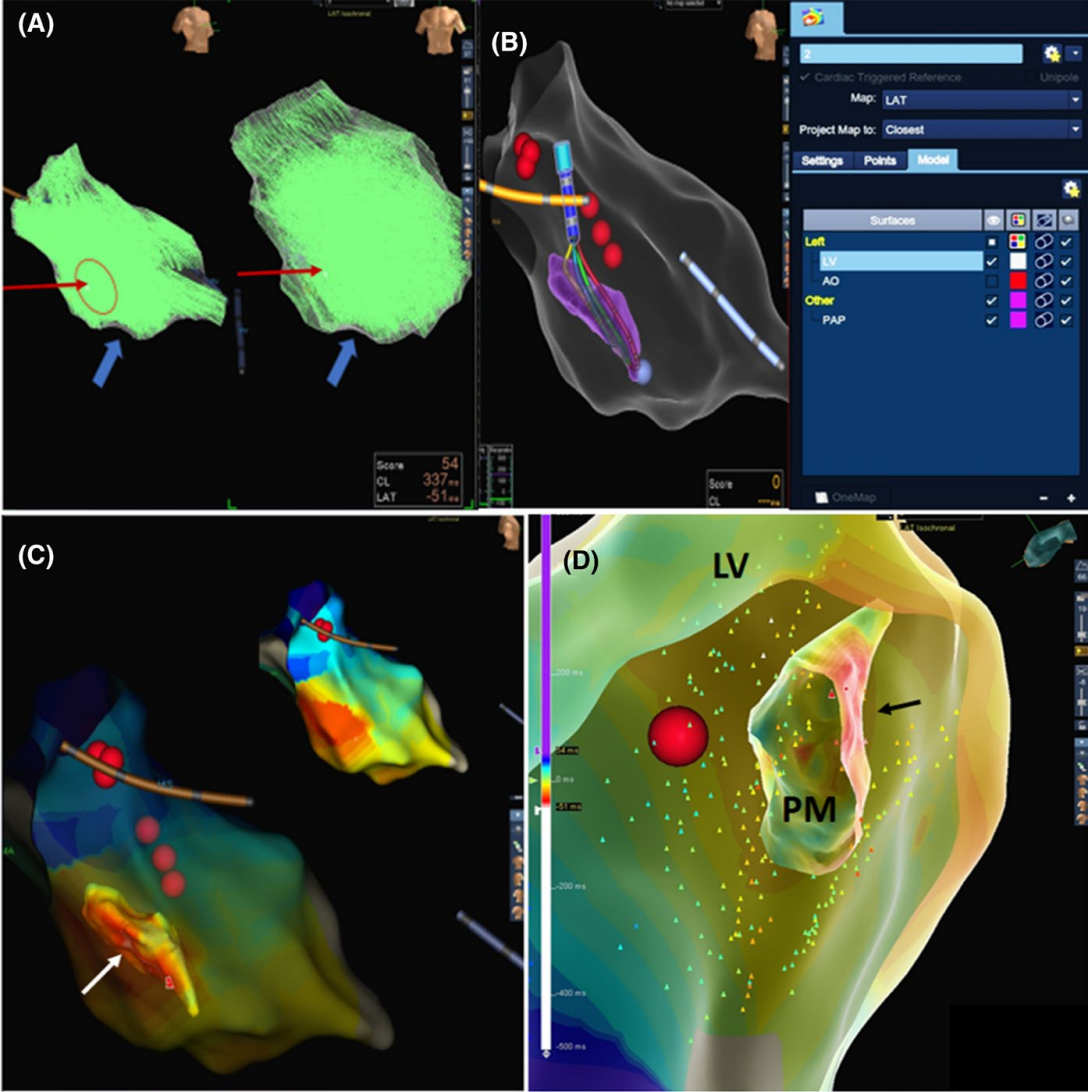
Illustration of the technique of deriving a PDX map. (A) The geometry fill is reduced to 10% and the “negative space” is seen (blue arrow) and identifies the probable location of the PM (red arrow) the inner geometrical points of which are responsible for the “negative space”. The site of earliest activation is then reconfirmed. These activation points will not be included by default to the activation color code, since they do not meet the criteria of interior projection algorithm of the EAM system. (B) After confirming these points, the corresponding geometry points are reassigned to another geometry surface with LV and PM represented as different surfaces. (C) Before reassigning the PM as another structure, the activation map will not project the earliest activation (white color) since it will not meet the interior projection criteria (upper, smaller geometry). After reassigning the PM as separate structure (lower, larger geometry), the activation map localizes the earliest activation (white color) to the inner structure(arrow). EAM algorithm is set as to project the activation points to the closest surface. (D) The “cut open” view of the LV as viewed from the mitral annular side showing the papillary muscle and the endocardial border. The site of earliest activation (white color) is clearly on the papillary muscle

After acquisition of a high‐density EAM, the surface fill is reduced to 10% so that the “negative space”, which represents the inner points originally excluded from the map, is seen (Figure 2). This is due to the inner points and represents the presence of an endocavitary structure within the shell. The surface fill is akin to the interpolation used in the activation map, and reducing it increases the resolution of the geometry. Adjusting the translucency allows to see the local activation points (and not the surface activation points) with early local activation that surround the negative space. Geometry points in these sites are then copied to a new surface model that corresponds to these internal structures, and the same activation map is applied to obtain a display of activation of the endocavitary structures.^1^

## CONFLICTS OF INTERESTS

None of the authors have any competing interests or conflicts of interests to declare.

## References

[1] Miller J, Dewland TA, Henrikson C, Reiss J, Patel A, Nazer B. Point Density eXclusion (PDX) electroanatomic mapping for ventricular arrhythmias arising from endocavitary structures. Hear Rhythm O2. 2020;1:394–8. 10.1016/j.hroo.2020.08.004 PMC8183858 34113896

